# Evidence in Urologic- and Pelvic-Surgery Research: Finding the IDEAL Way of Reporting

**DOI:** 10.1155/2017/2716759

**Published:** 2017-03-09

**Authors:** H. Gerullis, D. Barski, P. U. Malmström, X. Sun, T. H. Ecke

**Affiliations:** ^1^University Hospital for Urology, Klinikum Oldenburg, School of Medicine and Health Sciences, Carl von Ossietzky University Oldenburg, Oldenburg, Germany; ^2^Department of Urology, Lukas Hospital, Neuss, Germany; ^3^Department of Urology, Uppsala University, Uppsala, Sweden; ^4^University of Chengdu, Chengdu, China; ^5^Department of Urology, HELIOS Hospital, Bad Saarow, Germany

In 2009, the Idea, Development, Exploration, Assessment, Long-Term Follow-Up (IDEAL) Collaboration, an international, Oxford-based group of surgeons and methodologists, suggested a template of clear recommendations to define the fundamental stages of surgical innovations and related research [1–3]. IDEAL provides a framework for the evaluation of surgical innovations comparable to the existing standards for drug development. Ever since, the IDEAL recommendations have increasingly been applied in surgical research and reporting.

This special issue has been introduced with the aim of offering the possibility of publishing research results and particularly discuss them according to IDEAL to urologists and researchers connected to the field of urosurgery, urogynecology, surgery, and pelvic surgery.

While editing this special issue we have learned that the awareness towards the IDEAL recommendations is not as developed as one could hope. Although IDEAL has been applied in several prospective research projects and even shown applicable when retrospectively reporting the status of a surgical method or innovation it has not been used by the majority of submissions to this special issue. However, not labelling an innovative surgical or diagnostic method does not diminish its value as the recommendations have existed for 7 years only. This special issue aimed to contribute in divulging IDEAL and in encouraging surgeons and scientists to use these recommendations when reporting their innovations. As a conclusion it can be stated that the awareness of IDEAL needs to increase among both researchers and reviewers.

## References

[1] Barkun J. S., Aronson J. K., Feldman L. S. (2009). Evaluation and stages of surgical innovations. *The Lancet*.

[2] Ergina P. L., Cook J. A., Blazeby J. M. (2009). Challenges in evaluating surgical innovation. *The Lancet*.

[3] McCulloch P., Altman D. G., Campbell W. B. (2009). No surgical innovation without evaluation: the IDEAL recommendations. *The Lancet*.

